# Long-term consequences of cancer therapy: cognitive impairment following CAR T cell therapy

**DOI:** 10.1038/s41392-025-02324-6

**Published:** 2025-07-10

**Authors:** Kathrin Gabriel, Sebastian Kobold

**Affiliations:** 1 Division of Clinical Pharmacology, Department of Medicine IV, University Hospital, Ludwig-Maximilians Universität (LMU) of Munich, Munich, Germany; 2 German Cancer Consortium (DKTK), Partner Site Munich, Munich, Germany; 3 German Center for Lung Research (DZL), Partner Site Munich, Munich, Germany; 4 Einheit für Klinische Pharmakologie (EKLiP), Helmholtz Zentrum München, German Research Center for Environmental Health (HMGU), Neuherberg, Germany

**Keywords:** Cancer therapy, CNS cancer, Cancer models, Tumour immunology, Preclinical research

**Fig. 1:**
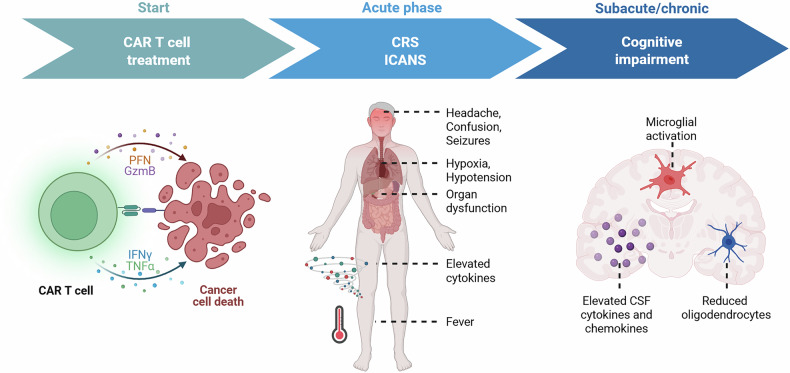
Temporal overview of onset and duration of side effects following administration of CAR T cell therapy. Compared to more acute toxicities such as CRS and ICANS, cognitive impairment can also be seen after months and years following CAR T cell administration. Underlying mechanisms are characterized by distinct neurobiological alterations such as elevated cerebrospinal fluid (CSF) cytokine and chemokine levels, reduced subcortical oligodendrocyte numbers, and microglial activation. Figure created in BioRender. G., K. (2025)

A recent publication by Geraghty et al. in *Cell* investigates how chimeric antigen receptor (CAR) T cell therapy can induce cognitive impairment in murine models of both central nervous system (CNS)- and non-CNS-based tumors.^1^ The authors identified persistent neuroinflammation as a key mechanism underlying these cognitive deficits and successfully explored novel therapeutic strategies, including microglial depletion and CCR3 blockade (Fig. 1).^1^

CAR T cell therapy has transformed treatment options for relapsed or refractory hematologic malignancies. Although clinical trials and long-term follow-up studies have confirmed its efficacy and an overall manageable acute toxicity profile, emerging evidence indicates that a subset of patients may develop persistent cognitive deficits post-treatment.^2^ While most encephalopathic symptoms resolve within the acute stage, typically a few weeks after treatment, a relevant number of patients suffer from long-lasting cognitive deficits such as impairments in memory, word-finding, and attention.^2^

Acute toxicities such as cytokine release syndrome (CRS) and immune effector cell-associated neurotoxicity syndrome (ICANS) are routinely monitored side effects of CAR T cell therapy. These are primarily driven by an overactivation of immune effector cells and elevated levels of proinflammatory cytokines such as TNF, IFN-γ, IL-1, and IL-6, resulting in endothelial dysfunction and disruption of the blood-brain barrier.^2^ In contrast, long-term cognitive side effects remain underrecognized and poorly understood, both in terms of incidence and mechanistic underpinnings. This limited understanding pertains also to therapeutic interventions. Current treatment regimens for CRS and ICANS include the use of corticosteroids and Tocilizumab, an antibody targeting the IL-6 receptor. However, there are no established interventions for the prevention or treatment of persistent cognitive impairment.^2^ Over the past years, cancer therapy-related cognitive impairment has gained recognition as a consequence of traditional oncological treatments, including chemotherapy and radiation. These therapies induce sustained neuroinflammation characterized by persistent activation of microglia and astrocytes, leading to reduced differentiation and depletion of oligodendroglial lineage cells.^3^

In this context, the recent study by Geraghty et al.^1^ investigates cognitive side effects of CAR T cell therapy. The authors employed multiple murine xenograft models of CNS and non-CNS tumors to demonstrate that tumor-clearing CAR T cell therapy can lead to deficits in attention and memory in mice.^1^ These findings were based on behavioral testing of attention and short-term memory (Novel Object Recognition Test) and spatial working memory (T-maze Test). Mice in the treatment groups receiving tumor-clearing doses of CAR T cells performed worse than their counterparts receiving mock-transduced or off-target-control CAR T cells. This was observed in a CNS-based model of diffuse intrinsic pontine glioma (DIPG), as well as in models of acute lymphoblastic leukemia and aggressive osteosarcoma.^1^ Notably, such deficits were not observed in an osteosarcoma tumor model that underwent rapid clearance with minimal inflammatory response, suggesting that detrimental effects may result from the induction of a broader and lasting immune response rather than direct CAR T cell-tumor interactions. The study further explored the underlying mechanisms of these cognitive impairments and identified persistent neuroinflammation as a key driver. Affected animals were characterized by reactive microglial phenotypes in white matter regions, elevated microglial chemokine expression, and increased concentrations of cerebrospinal fluid cytokines and chemokines.^1^ Among these, CCL11/Eotaxin, CCL2, CCL7 and CXCL10 were notably elevated. Microglial cells themselves showed a shift to a reactive transcriptional state with upregulated chemokines like CCL11, CCL19, and CCL24. The resulting immune activation leads to dysregulation of oligodendroglial cells and impaired hippocampal neurogenesis, marked by reductions in subcortical oligodendrocyte numbers and decreased myelinated axon density.^1^ For the CNS-based DIPG model, similar effects were observed when CAR T cells were administered intracranially rather than intravenously, demonstrating the relevance of these findings across therapeutic settings. The authors validated their findings using single-nucleus RNA sequencing of frontal lobe samples from patients who received CAR T cell treatment for brainstem tumors. The analysis revealed reactive transcriptional states in microglia and oligodendrocytes consistent with findings in the murine models.^1^ Additionally, the study explored therapeutic avenues by targeting specific elements of the inflammatory cascade. Both microglial depletion using the CSF1R inhibitor PLX5622 and blockade of the chemokine receptor CCR3 rescued oligodendroglial and microglial pathology and improved cognitive performance. In contrast, Anakinra, an IL-1 receptor antagonist used clinically to treat neurotoxicities such as ICANS, did not restore cognitive function.^1^ These results support a causal role of the identified immune pathways in driving cognitive impairment and highlight the relevance of the investigated therapeutic strategies. However, most of the study’s evidence was derived from immunodeficient xenograft models, which do not fully recapitulate the complexity of human systemic immune responses. The only immunocompetent model employed, a melanoma system, could not be assessed for cognitive outcomes due to tumor-associated pain. Nevertheless, since lymphodepleting chemotherapy has been shown to impair cognition, the use of immunocompromised animals allows the attribution of cognitive effects specifically to CAR T cell therapy.^1^ Another open question relates to other clinically relevant CAR constructs. BCMA is a target antigen for two FDA-approved CAR T cell products, and it remains unclear whether the findings also apply in that context. This is particularly relevant in light of recent clinical observations of cognitive side effects, including Parkinsonism, following BCMA-targeted CAR T cell treatment.^4^

The study by Geraghty et al. extends the framework of CAR T cell-associated toxicities from acute syndromes such as CRS and ICANS towards a form of delayed cognitive impairment that arises independently of acute neurotoxicity. The identification of distinct mechanisms, such as persistent neuroinflammation associated with oligodendroglial dysfunction and disrupted hippocampal neurogenesis, underlines the need for further research to better understand and treat such sequelae. These findings may even extend beyond the field of oncology. Post-infectious syndromes have shown the vulnerability of neurological homeostasis to peripheral immune activation, where white matter microglial responses and myelin dysregulation are associated with cognitive impairment after COVID-19 infection, suggesting potentially shared pathways between these cases and patients that experience neurotoxicities after immunotherapy.^5^ In light of such emerging evidence, considerations of cognitive side effects of immunotherapy should be included in therapeutic risk-benefit assessments, and additional research should be dedicated to developing strategies to mitigate these long-term consequences.
